# Quantum coherence and quantum phase transitions

**DOI:** 10.1038/srep26365

**Published:** 2016-05-19

**Authors:** Yan-Chao Li, Hai-Qing Lin

**Affiliations:** 1 College of Materials Science and Opto-Electronic Technology, University of Chinese Academy of Sciences, Beijing 100049, People’s Republic of China; 2 Beijing Computational Science Research Center, Beijing 100193, People’s Republic of China

## Abstract

We study the connections between local quantum coherence (LQC) based on Wigner-Yanase skew information and quantum phase transitions (QPTs). When applied on the one-dimensional Hubbard, *XY* spin chain with three-spin interaction, and Su-Schrieffer-Heeger models, the LQC and its derivatives are used successfully to detect different types of QPTs in these spin and fermionic systems. Furthermore, the LQC is effective as the quantum discord (QD) in detecting QPTs at finite temperatures, where the entanglement has lost its effectiveness. We also demonstrate that the LQC can exhibit different behaviors in many forms compared with the QD.

Quantum phase transitions (QPTs)^1^, which are purely driven by quantum fluctuations, occur at absolute zero temperature. QPTs are caused by the changing driving Hamiltonian parameters, such as an external magnetic field or the coupling constant. One QPT marked by a quantum critical point (QCP) corresponds to an abrupt change in the ground state, which results in qualitatively distinct properties of the matter. Although QPTs occur at absolute zero temperature, they can also be detected under extremely low temperatures where thermal fluctuations are not adequately strong to excite the ground state, and the system remains dominated by quantum fluctuations. A study on QPTs can help us to understand the physical properties of various matters from the quantum mechanics perspective. Many unsolved puzzles in physics, such as heavy-fermion metals and high-temperature superconductors^2^,^3^,^4^, are suspected to be understood by investigating QPTs. The finding of magnetically mediated superconductivity in a heavy-fermion system^5^, furthermore, enhances the interest in exploring QPT investigation.

In recent years, the relation between QPTs and quantum information has caused great attention, and several concepts borrowed from quantum information science are successfully used in the study on QPTs^6^,^7^,^8^,^9^,^10^,^11^,^12^,^13^,^14^,^15^; these concepts include entanglement^8^,^9^, mutual information^10^, quantum fidelity^12^,^14^, and quantum discord (QD)^15^,^16^,^17^. Very recently, the relationship between QPTs and the ground-state local convertibility is investigated in ref. 18, where the authors pointed out that the local convertibility can be used to detect QPTs. No a priori knowledge of the order parameter and the symmetry of the system is required. Thus, these concepts can be applied conveniently and effectively to detect QPTs on different systems, thereby making them potential universal criteria in the characterization of QPTs. Quantum discord in particular, which is a measure of quantum correlations beyond entanglement, has been demonstrated as an effective detector of critical points of QPTs at finite temperatures, where the entanglement of formation and other thermodynamic quantities have lost their ability to detect QPTs because of thermal fluctuations^15^,^17^. Given that this remarkable property of QD may improve the application for detecting QPTs in experiments, QD attracts considerable research interest^19^,^20^.

However, similar to one definition of quantum correlation, the Wigner-Yanase skew information (WYSI) is rarely studied to detect QPTs. WYSI, which quantifies the amount of information contained in a quantum state, reflects the information of a state skewed to an observable^21^ or the quantum uncertainty of an observable in a quantum state^22^,^23^. WYSI has been proven to satisfy all the criteria for coherence monotones^24^ and hence can be used as an efficient measure to quantify quantum coherence (QC). A simplified alternative version is introduced in ref. 25. This version is the lower bound of the WYIS, and it is meaningful in quantifying QC. Furthermore, it can be measured in an interferometric setup only using two programmable measurements. Recently, Karpat *et al.* explored the link between QPTs and the WYSI. They pointed out that the skew information supplies the necessary information to reveal the occurrence of a phase transition^26^. However, They focus only on the QPT of the *XY* model. A comprehensive understanding of the relationship between the WYSI and QPTs, especially the effectiveness and universality of the WYSI in detecting QPTs, is desired. Therefore, in this study, we scrutinize the WYSI on three systems with different types of QPTs. Through a comparison with the entanglement of formation (EOF) and QD, we aim to acquire a comprehensive understanding of quantum coherence in identifying QPTs.

## Quantum Coherence, Quantum Discord, and Entanglement of Formation

### Quantum coherence

WYSI, which satisfies the criteria for coherence monotones^22^,^24^, is a reliable measure of coherence^25^. The *K* coherence of a quantum state can be written as


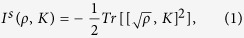


where *ρ* is the density matrix of a quantum state, *K* is an observable, and [...] denotes the commutator. Considering he Hermiticity of 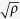 and *K*, their commutator is skew Hermitean, and the square of the commutator is Hermitean and negative semidefinite. Therefore, *I*^*s*^ is always positive except when *ρ* and *K* commute, in which case *I*^*s*^ = 0. *I*^*s*^ is initially introduced to measure the information embodied in a state skewed to an observable^21^. Recently, the WYSI *I*^*s*^ has been proven to satisfy all the criteria for coherence monotones, and thus, it can be used as an efficient measure to quantify QC^24^,^25^. For a two-site subsystem, that is, *A* and *B*, if we choose the observable at *A*, then *K* is written as 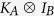. Thus, *I*^*s*^ is written as 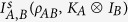, which quantifies the QC between *A* and *B*. In addition, *I*^*s*^ has a simplified alternative version


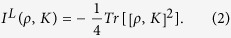


This version is the lower bound of *I*^*s*^. Given that *ρ* has no square root terms, this version is a function of observable, thereby making it relevant to experimental measurements. Moreover, the lower bound of *I*^*s*^ has been used to detect QPTs in the Ising model in ref. 26. Therefore, in the present study, we focus on this simplified version to further investigate its relationship with QPTs.

### Quantum discord

The mutual information between two arbitrary parts *A* and *B* has two different expressions: 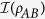 and 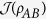. In a classical case, the expressions are equivalent. However, in the quantum domain, they are not equal. The minimum difference between them is defined as the QD^17^,^27^,^28^: 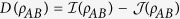, where 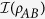 measures the total correlation, whereas 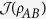 corresponds to the classical correlation of state *ρ*_*AB*_ in quantum information theory (*ρ*_*AB*_ is the reduced-density operator of *A* and *B*). The expressions can be written as^15^,^28^,^29^


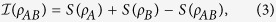



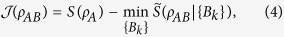


with the von Neumann entropy 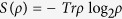, and 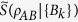 is the measurement-induced quantum conditional entropy, which is a measure of how uncertain is *A* when *B* is known. After measuring *B*, the quantum state *ρ*_*AB*_ changes to 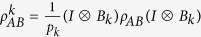, with *I* as the identity operator for *A* and *p*_*k*_ = *Tr* 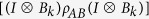. Then the measurement-induced quantum conditional entropy can be written as 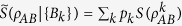. Its minimum 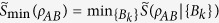 is achieved from a complete set of projective measures {*B*_*k*_} on part *B*. For a two-spin subsystem, the projectors 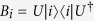, where 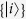 is the standard basis 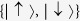, and the transform matrix *U* is parameterized as^29^


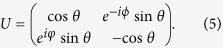


Through changing *θ* and *φ*, the minimum of the measurement-induced condition entropy 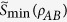 can be determined and then QD can be obtained. Researchers believe that QD measures the quantumness of correlations.

### Entanglement of formation

Concurrence 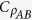, which is one definition of entanglement, measures the quantum entanglement between *A* and *B*. It can be written as 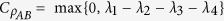, where *λ*_1_, *λ*_2_, *λ*_3_, and *λ*_4_ are the square roots of the eigenvalues of 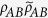 in descending order, 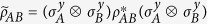 is the time-reversed matrix of *ρ*_*AB*_, 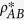 is the complex conjugation of *ρ*_*AB*_, and *σ*^*y*^ denotes the *y* component of Pauli operator. The EOF is defined as^15^,^17^,^30^


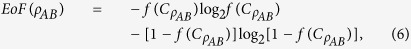


with 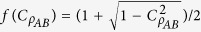, which is a monotonically increasing function of the concurrence. Thus, EOF satisfies the criteria for entanglement monotone. We choose the EOF as a measure of entanglement to compare our results for QD and QC.

## Results and Discussions

### Bow state in the one-dimensional half-filed extended hubbard model

We first consider the one-dimensional half-filled extended Hubbard model (EHM). The Hamiltonian for the EHM reads


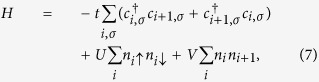


where 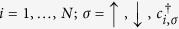 and *n*_*i*_ are the creation and number operators at site *i*, respectively, *t* is the hopping integral (*t* = 1 is set as the energy unit here), and *U* and *V* are the on-site and the nearest-neighbor Coulomb interactions, respectively. This model has been studied extensively, and its ground-state diagram typically includes the charge-density wave (CDW), spin-density wave (SDW), phase separation (PS), singlet (SS), and triplet superconducting phases (TS)^9^,^31^,^32^,^33^. However, a controversy focused on a narrow strip–the supposed bond-order-wave (BOW) state–in the repulsive regime along 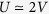 line for weak couplings. At the beginning, this region was regarded as a direct transition between the SDW and CDW phases^31^,^34^,^35^,^36^,^37^. However, ref. 33 pointed out that an intermediated BOW state was detected in a narrow strip between the SDW and CDW phases in weak couplings. This point was supported by numerical results, such as the Monte Carlo calculations^38^,^39^ and density-matrix renormalization group (DMRG) methods^40^. However, there are also different conclusions^41^,^42^. Therefore, in this subsection, we mainly focus on this region.

We calculate the local two-site QC based on the WYSI. *ρ*_*AB*_ here is the reduced density matrix for two neighboring sites *A* and *B* in the chain. We take the number of electron with spin up *n*_*A*↑_ on site *A* as the observable *K*_*A*_. On the basis spanned by 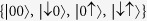, *K*_*A*_ can be written as


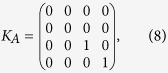


where the four states in the basis refer to the four possibilities that site *A* is either unoccupied, occupied by a particle of spin down, a particle of spin up, or doubly occupied, respectively. If the reduced density matrix *ρ*_*AB*_ is known, then the local quantum coherence based on 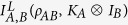 in Eq. 2 is available. Later in the study, we use the DMRG technique with anti-periodic boundary condition to obtain the ground state and the reduced-density matrix to calculate the QC.

**Figure 1:**
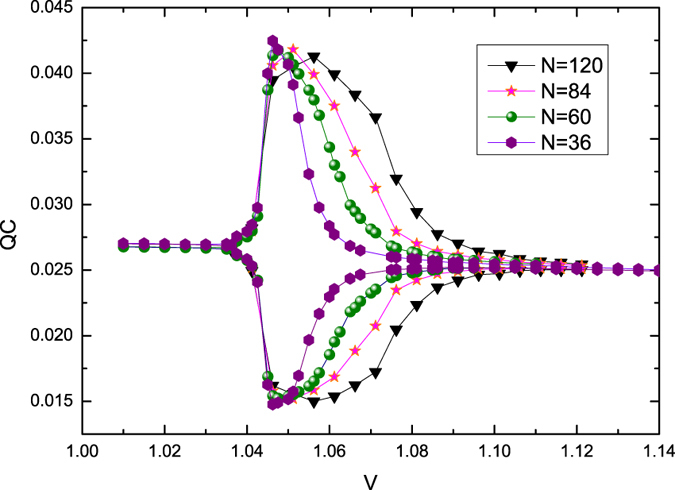
Two-site QC 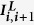 for *i* = *N*/2 and *i* = *N*/2 + 1 of the EHM for *U* = 2.0 under different chain lengths.

The QC as a function of *V* at *U* = 2.0 under different system-size *N* is plotted in Fig. 1. For a given *N*, two neighboring two-site QCs 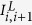 (here *A* = *i* and *B* = *i* + 1) for *i* = *N*/2 and *i* = *N*/2 + 1 are calculated, respectively. Three regimes can be clearly distinguished. In the range −1.035 < *V* < 1.1, 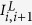 for *i* = *N*/2 and *i* = *N*/2 + 1 are different, and the difference becomes clearer as *N* increases, which reflects that the two pair sites are coupled by strong and weak bounds. Therefore, the state in this regime is dimerized, and it should be the BOW state. The other two regimes at the left and right sides of this region should be the SDW and CDW states according to the known phase diagrams of the EHM^9^,^33^, respectively. The local QC here clearly indicates the BOW state in this fermionic system.

### Phase transitions in the *XY* model with three spin interactions

The *XY* spin model in a transverse field with three-spin interaction (XYT) is defined by the Hamiltonian


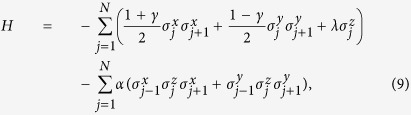


where *σ*^*α*^ (*α* = *x*, *y*, *z*) are the Pauli matrices, *N* is the spin numbers of the chain, *γ* is the anisotropy parameter, *λ* denotes the external magnetic field, and *α* describes the three-spin interactions.

Introducing the Jordan-Wigner^1^, Fourier, and Bogoliubov transformations ensures that *H* can be exactly diagonalized in momentum space (ref. 17). Then the finite-temperature reduced density matrix *ρ*_*i*,*j*_ for two neighboring spins *i* and *j* can be obtained as^43^,^44^


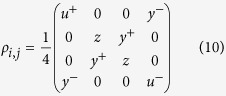


where the elements are related to the correlation functions


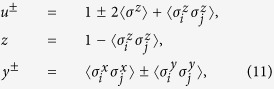


where the mean magnetization and the two-point correlation functions are calculated as^45^,^46^


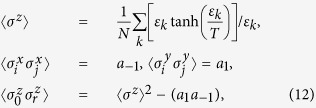


where 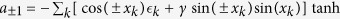 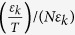, 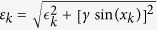 with 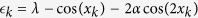 and *x*_*k*_ = 2*πk*/*N* is the energy spectrum, and *T* is temperature. Now that *ρ*_*i*,*j*_ is available, the QC related to *ρ*_*i*,*j*_ can be calculated directly. In addition, the EoF and QD are calculated for comparison.

**Figure 2:**
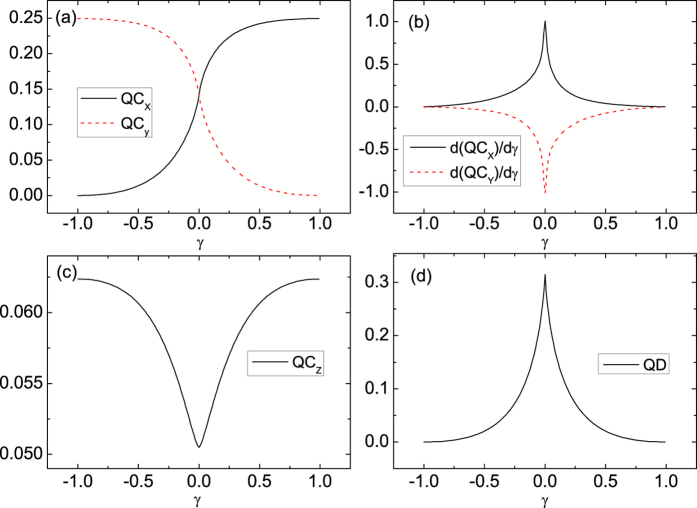
(**a**) *σ*_*x*_ and *σ*_*y*_ coherences, (**b**) their first derivatives with respect to *γ*, (**c**) *σ*_*z*_ coherence, and (**d**) the QD as a function of *γ* at *λ* = *α* = 0 for *N* = 1001.

The two-site local *σ*_*β*_ as a function of *γ* at *λ* = *α* = 0 are plotted in Fig. 2, where the footnotes *β* = *x*, *y*, *z* denote observables in different directions. The *σ*_*x*_ and *σ*_*y*_ coherences do not peak at the first-order phase transition point at *γ* = 0, but they show symmetry at the critical point and cross at *γ* = 0 as shown in Fig. 2(a). These phenomena are easily understood. The negative and positive values of *γ* actually reflect different intensities of interaction between two spins along *x* and *y* directions, and the intensities along different directions are symmetrical near *γ* = 0. Therefore, the coherence along *x* direction should be increasing monotonically and symmetrical near *γ* = 0 with the *σ*_*y*_ coherence. Although the *σ*_*x*_ and *σ*_*y*_ coherences do not exhibit any divergences, the divergences of their first derivative with respect to *γ* spotlight the critical point at *γ* = 0. This behavior differs from most indicators of QPT, such as entanglement and QD. At this first-order transition point, they would show divergence by themselves instead of their first derivatives, e.g. the behavior of QD in Fig. 2(d). In addition, given the lack of influence of anisotropy, the local *σ*_*z*_ coherence is divergent in the QCP as shown in Fig. 2(c). At the same time, the variational trend of the value of *σ*_*z*_ is contrary to that of QD—the *σ*_*z*_ coherence exhibits a valley, whereas the QD displays a peak at the QCP. These different behaviors can be interpreted as follows: the *σ*_*z*_ coherence reflects only the coherence along one direction, whereas the QD contains quantum correlations from various directions, thereby resulting in the difference in their behaviors.

**Figure 3:**
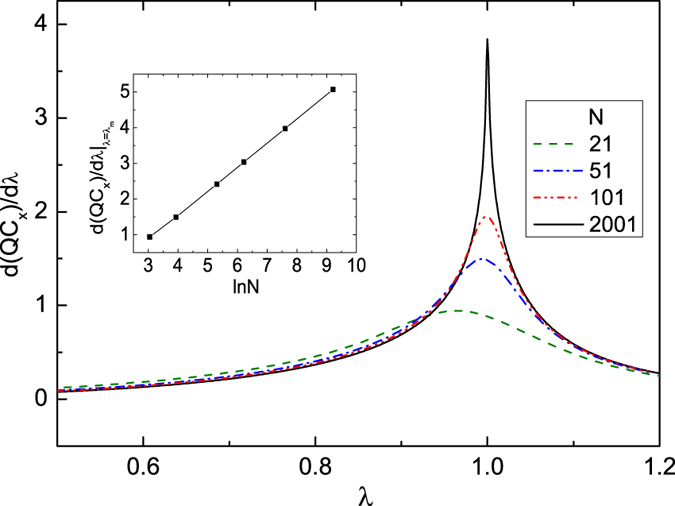
First derivative of the *σ*_*x*_ coherence *d*(*QC*_*x*_)/*dλ* under different system sizes *N* at *γ* = 0.5 and *α* = 0.0. The inset shows the finite-size scaling behavior of the maximum in *d*(*QC*_*x*_)/*dλ*.

We then consider the second-order phase transition at *λ* = 1.0. The first derivatives of the *σ*_*x*_ coherence with respect to *λ* under different system sizes are shown in Fig. 3. A peak is observed near at the critical point *λ* = 1.0, and the peak is pronounced as *N* increases. The size-dependent scaling behavior of the peak indicates that it will be divergent in the thermodynamic limit (inset Fig. 3). Therefore, the *σ*_*x*_ coherence here indicates the second-order phase transition. In addition, the *σ*_*y*_ and *σ*_*z*_ coherences exhibit similar behaviors (we do not show them here) and can be used to detect the critical point.

**Figure 4:**
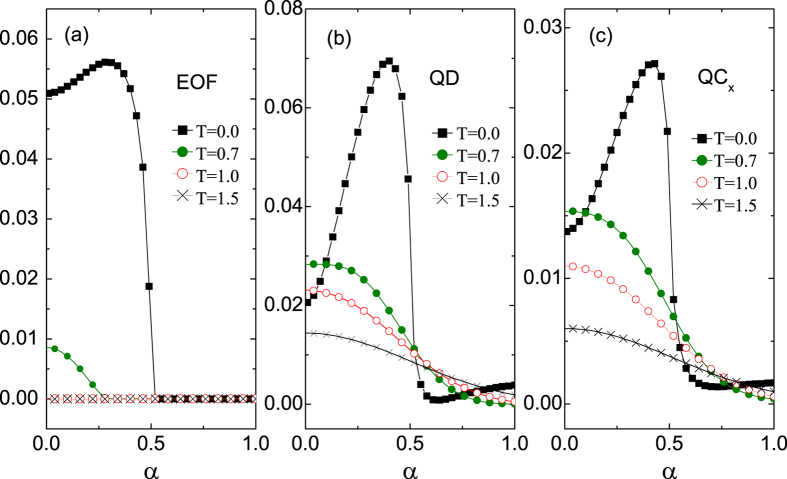
(**a**) The EOF, (**b**) QD, and (**c**) *QC*_*x*_ as a function of *α* under different temperatures at *λ* = 0.0 and *γ* = 0.5 with *N* = 1001.

**Figure 5:**
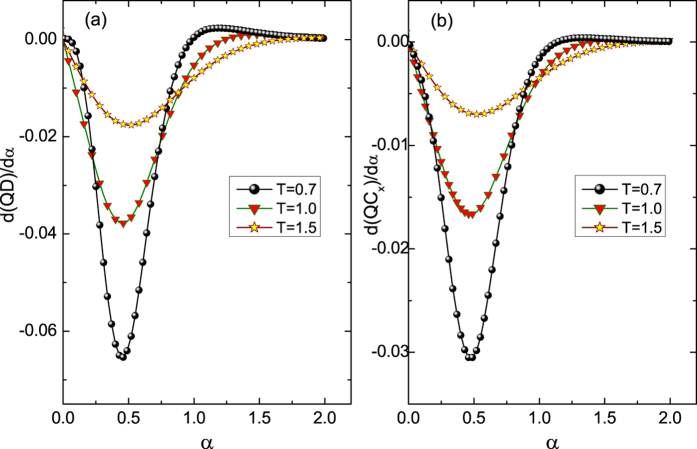
(**a**) *d*(*QD*)/*dα* and (**b**) *d*(*QC*_*x*_)/*dα* as a function of *α* under different temperatures at the same condition as that in Fig. 4.

After demonstrating that the QC can be used to indicate the QPTs at absolute zero temperature, we further consider its performance at finite temperatures. It has been pointed out that there is a second order phase transition at *α* = 0.5 for *γ* = 0.5 and *λ* = 0.0. The EOF, QD, and QC as a function of *α* under different temperatures are illustrated in Fig. 4. The EOF tends to be zero after *T* > 1.0 for all values of *α* and thus can not indicate this transition. However, the QD and QC, which have similar behaviors, do not equal zero for all *α* values even at extremely high temperature (e.g., *T* = 2.5). Their first derivatives with respective to *α* under different temperatures are shown in Fig. 5. The valley structure that reflects the phase transition does not dematerialize at extremely high temperatures. Moreover, the temperature that corresponds to the dematerialized valley of the *σ*_*x*_ coherence is as high as that of the QD. Therefore, the QC has a similar ability to indicate QPTs at finite temperatures.

### Qc and topological phase transition in the ssh model

The one-dimensional Su-Schrieffer-Heeger (SSH) model is proposed for polyacetylene^47^. Its Hamiltonian is written as


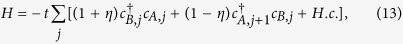


where *A* and *B* are the sublattice indices, *η* denotes the dimerization, and *t* is the transfer integral (here *t* = 1). The sublattice symmetry between the *A* and *B* sublattices results in particle-hole symmetry. Given the sublattice symmetry, a topological induce, which equals the number of zero-energy states, can be defined^47^. A topological phase transition at *η* = 0 exists in the system.

**Figure 6:**
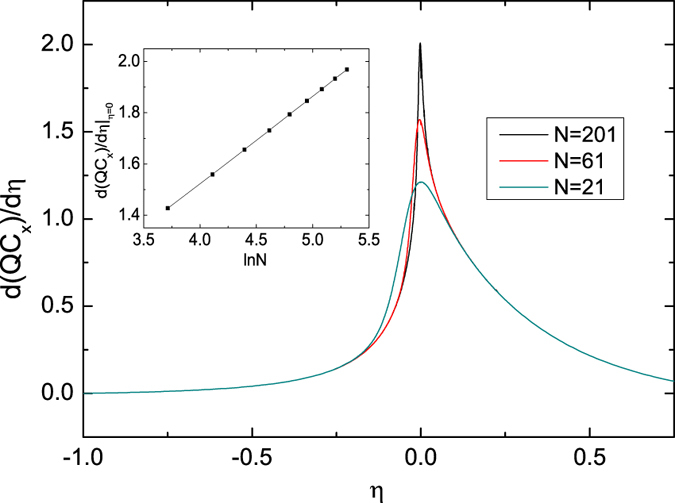
*d*(*QC*_*x*_)/*dη* as a function of *η* under different system sizes *N*. The finite-size scaling behavior of *d*(*QC*_*x*_)/*dη* at the topological critical point *η* = 0 is shown as an inset.

Using the DMRG method, we calculate the two-sublattice *σ*_*x*_ coherence. The reduced-density matrix for the calculations of the *σ*_*x*_ coherence is derived for the two sublattices *A* and *B* at the same site *j* = *N*/2 of the chain. The first derivative of *σ*_*x*_ coherence with respect to *η* peaks near *η* = 0.0, which is the critical point of the topological transition, and it is pronounced as *N* increases (Fig. 6). The value of the peak will be divergent at the thermodynamic limit (see the inset of Fig. 6). Therefore, we can conclude that the two-sublattice *σ*_*x*_ coherence here successfully characterizes the topological phase transition.

#### Summary

On the basis of the lower bound of the WYSI, QC is investigated on different systems (i.e., fermionic system, spin system, and the SSH model with a topological phase transition). Our results show that the dimerized property of the BOW state of the fermionic Hubbard model can be clearly demonstrated by two neighboring QCs. For the XYT model, we find that both the first- and continuous-order transitions are efficiently detected by the first derivative of the QC at zero temperature. The behavior of the first derivative of QC rather than the actual QC peaks at the first-order QCP. This behavior differs from that of most QPT indicators. We conclude that this novel phenomenon is caused by the anisotropy of the system and the directivity of the observable selected for the calculation of QC. Furthermore, compared with entanglement, the QC can exist at an extremely high temperature, and its first derivative can reflect the undergoing of QPTs. This ability of QC is as good as that of QD. Finally, the topological quantum phase transition in the SSH model can also be characterized by the QC.

## Additional Information

**How to cite this article**: Li, Y.-C. and Lin, H.-Q. Quantum coherence and quantum phase transitions. *Sci. Rep.* **6**, 26365; doi: 10.1038/srep26365 (2016).

## References

[b1] Sachdev S. Quantum phase transition. Cambridge University Press, Cambridge, UK (1999).

[b2] Gegenwart P., Si Q. & Steglich F. Quantum criticality in heavy-fermion metals. Nature Physics 4, 186–197 (2008).

[b3] Zhu L., Garst M., Rosch A. & Si Q. Universally diverging Grüneisen parameter and the magnetocaloric effect close to quantum critical points. Phys. Rev. Lett. 91, 066404 (2003). 12935092 10.1103/PhysRevLett.91.066404

[b4] Küchler R. *et al.* Divergence of the Grüneisen ratio at quantum critical points in heavy fermion metals. Phys. Rev. Lett. 91, 066405 (2003). 12935093 10.1103/PhysRevLett.91.066405

[b5] Mathur N. D. *et al.* Magnetically mediated superconductivity in heavy fermion compounds. Nature 394, 39–43 (1998).

[b6] Zhu S. L. Scaling of geometric phases close to the quantum phase transition in the XY spin chain. Phys. Rev. Lett. 96, 077206 (2006). 16606137 10.1103/PhysRevLett.96.077206

[b7] Chen S., Wang L., Hao Y. J. & Wang Y. P. Intrinsic relation between ground-state fidelity and the characterization of a quantum phase transition. Phys. Rev. A 77, 032111 (2008).

[b8] Osterloh A., Amico L., Falci G. & Fazio R. Scaling of entanglement close to a quantum phase transition. Nature 416, 608–610 (2002). 11948343 10.1038/416608a

[b9] Gu S. J., Deng S. S., Li Y. Q. & Lin H. Q. Entanglement and quantum phase transition in the extended Hubbard model. Phys. Rev. Lett. 93, 086402 (2004). 15447208 10.1103/PhysRevLett.93.086402

[b10] Cui J., Cao J. P. & Fan H. Quantum-information approach to the quantum phase transition in the Kitaev honeycomb model. Phys. Rev. A 82, 022319 (2010).

[b11] Cui J. *et al.* Quantum phases with differing computational power. Nature Commun. 3, 812 (2012). 22549841 10.1038/ncomms1809

[b12] Zanardi P. & Paunkovic N. Ground state overlap and quantum phase transitions. Phys. Rev. E 74, 031123 (2006). 10.1103/PhysRevE.74.031123 17025610

[b13] Zanardi P., Quan H. T., Wang. X. G. & Sun C. P. Mixed-state fidelity and quantum criticality at finite temperature. Phys. Rev. A 75, 032109 (2007).

[b14] Quan H. T., Song Z., Liu X. F., Zanardi P. & Sun C. P. Decay of Loschmidt echo enhanced by quantum criticality. Phys. Rev. Lett. 96, 140604 (2006). 16712060 10.1103/PhysRevLett.96.140604

[b15] Werlang T., Trippe C., Ribeiro G. A. P. & Rigolin G. Quantum correlations in spin chains at finite temperatures and quantum phase transitions. Phys. Rev. Lett. 105, 095702 (2010). 20868176 10.1103/PhysRevLett.105.095702

[b16] Liu S. Y., Zhang Y. R., Zhao L. M., Yang W. L. & Fan H. General monogamy property of global quantum discord and the application. Ann. Phys. 348, 256–269 (2014).

[b17] Li Y. C. & Lin H. Q. Thermal quantum and classical correlations and entanglement in the XY spin model with three-spin interaction. Phys. Rev. A 83, 052323 (2011).

[b18] Liu S. Y. *et al.* Phase diagram of quantum critical system via local convertibility of ground state. Preprint at http://arxiv.org/abs/1510.07115 (2015). 10.1038/srep29175 PMC4933908 27381284

[b19] Huang Y. C. Scaling of quantum discord in spin models. Phys. Rev. B 89, 054410 (2014).

[b20] Liu S. Y., Zhang Y. R., Yang W. L. & Fan H. Global quantum discord and quantum phase transition in XY model. Ann. Phys. 362 805–813 (2015).

[b21] Wigner E. P. & Yanase M. M. Information contents of distributions. Proc. Natl. Acad. Sci. USA 49, 910–918 (1963). 16591109 10.1073/pnas.49.6.910 PMC300031

[b22] Luo S. Heisenberg uncertainty relation for mixed states. Phys. Rev. A 72, 042110 (2005).

[b23] Luo S. Brukner-Zeilinger invariant information. Theor. Math. Phys. 151, 693 (2007).

[b24] Baumgratz T., Cramer M. & Plenio M. B. Quantifying coherence. Phys. Rev. Lett. 113, 140401 (2014). 25325620 10.1103/PhysRevLett.113.140401

[b25] Girolami D. Observable measure of quantum coherence in finite dimensional systems. Phys. Rev. Lett. 113, 170401 (2014). 25379903 10.1103/PhysRevLett.113.170401

[b26] Karpat G., Çakmak B. & Fanchini F. F. Quantum coherence and uncertainty in the anisotropic XY chain. Phys. Rev. B 90, 104431 (2014).

[b27] Ollivier H. & Zurek W. H. Quantum discord: a measure of the quantumness of correlations. Phys. Rev. Lett. 88, 017901 (2001). 11800986 10.1103/PhysRevLett.88.017901

[b28] Henderson L. & Vedral V. Classical, quantum and total correlations. J. Phys. A: Math. Gen. 34, 6899–6905 (2001).

[b29] Sarandy M. S. Classical correlation and quantum discord in critical systems. Phys. Rev. A 80, 022108 (2009).

[b30] Maziero J., Guzman H. C., Céleri, Sarandy M. S. & Serra R. M. Quantum and classical thermal correlations in the XY spin-1/2 chain. Phys. Rev. A 82, 012106 (2010).

[b31] Lin H. Q., Campbell D. K. & Clay R. T. Broken symmetries in the one-dimensional extended Hubbard model. Chin. J. Phys. 38, 1 (2000).

[b32] Nakamura M. Mechanism of CDW-SDW transition in one dimension. J. Phys. Soc. Jpn. 68, 3123–3126 (1999).

[b33] Nakamura M. Tricritical behavior in the extended Hubbard chains. Phys. Rev. B 61, 16377 (2000).

[b34] Hirsch J. E. Charge-density-wave to spin-density-wave transition in the extended Hubbard model. Phys. Rev. Lett. 53, 2327 (1984).

[b35] Lin H. Q. & Hirsch J. E. Condensation transition in the one-dimensional extended Hubbard model. Phys. Rev. B 33, 8155 (1986). 10.1103/physrevb.33.8155 9938207

[b36] Voit J. Phase diagram and correlation functions of the half-filled extended Hubbard model in one dimension. Phys. Rev. B 45, 4027 (1992). 10.1103/physrevb.45.4027 10002015

[b37] Zhang G. P. Ground-state phase diagram of the one-dimensional extended Hubbard model: A density-matrix renormalization-group approach. Phys. Rev. B 56, 9189 (1997).

[b38] Sengupta P., Sandvik A. W. & Campbell D. K. Bond-order-wave phase and quantum phase transitions in the one-dimensional extended Hubbard model. Phys. Rev. B 65, 155113 (2002).

[b39] Sandvik A. W., Sengupta P. & Campbell D. K. Comment on “Ground-state phase diagram of a half-filled one-dimensional extended Hubbard model”. Phys. Rev. Lett. 91, 089701 (2003). 14525284 10.1103/PhysRevLett.91.089701

[b40] Zhang Y. Z. Dimerization in a half-filled one-dimensional extended Hubbard model. Phys. Rev. Lett. 92, 246404 (2004). 15245116 10.1103/PhysRevLett.92.246404

[b41] Zhang G. P. Accurate ground-state phase diagram of the one-dimensional extended Hubbard model at half filling. Phys. Rev. B 68, 153101 (2003).

[b42] Jeckelmann E. Ground-state phase diagram of a half-filled one-dimensional extended Hubbard model. Phys. Rev. Lett. 89, 236401 (2002). 12485022 10.1103/PhysRevLett.89.236401

[b43] Wang X. G. Entanglement and spin squeezing in the three-qubit transverse Ising model. Phys. Lett. A 331, 164–169 (2004).

[b44] Gu S. J., Sun C. P. & Lin H. Q. Universal role of correlation entropy in critical phenomena. J. Phys. A: Math. Theor. 41, 025002 (2008).

[b45] Barouch E. & McCoy B. M. Statistical mechanics of the XY model. I. Phys. Rev. A 2, 1075 (1970).

[b46] Barouch E. & McCoy B. M. Statistical mechanics of the XY model. II. spin-correlation functions. Phys. Rev. A 3, 786 (1971).

[b47] Wakatsuki R., Ezawa M., Tanaka Y. & Nagaosa N. Fermion fractionalization to Majorana fermions in a dimerized Kitaev superconductor. Phys. Rev. B 90, 014505 (2014).

